# Enigma of cerebrospinal fluid dynamics

**DOI:** 10.3325/cmj.2014.55.287

**Published:** 2014-08

**Authors:** Marijan Klarica, Darko Orešković

**Affiliations:** 1 Department of Pharmacology and Croatian Institute for Brain Research, University of Zagreb School of Medicine, Zagreb, Croatia; 2 Department of Molecular Biology, Ruđer Bošković Institute, Zagreb, Croatia

## Two hypotheses on CSF physiology

Cerebrospinal fluid (CSF) is a major part of the central nervous system (CNS) extracellular fluid, and fine regulation of its composition is vital to the brain’s health. Although CSF dynamics has been studied for an entire century, many of its aspects are still insufficiently understood. Today there are two hypotheses (1,2) on CSF physiology: a) traditional hypothesis and b) microcirculatory/microvessel hypothesis.

According to the traditional hypothesis, CSF is formed inside the brain ventricles, mostly by secretion from the choroid plexuses, and it circulates along the ventricles and subarachnoid space to be absorbed across the arachnoid villi into the dural venous sinuses, and/or viacranial and spinal nerves paraneural sheaths into the lymphatic system. Since substance exchange occurs between the CNS extracellular interstitial fluid (ISF) and CSF, it is assumed that CSF serves as a sink for the removal of various metabolites out of the CNS by its unidirectional pulsatile flow and absorption (3-5). This traditional hypothesis, with minor modifications, represents a common point of reference in scientific papers, review articles, and textbooks on the issue (6,7). Additionally, this hypothesis has been used to explain the yet clinically unsolved pathological states such as increase in intracranial pressure and hydrocephalus.

The emerging concept of CSF physiology described as microcirculatory/microvessel hypothesis suggests that CNS microvessels are instrumental in fluid filtration and reabsorption inside the brain and spinal cord parenchyma, as well as inside the CSF system (2,8-10). It seems that the osmotic pressure change inside the CNS capillary network is crucial in regulation of ISF and CSF volumes, which are continuously mixed by to-and-fro fluid pulsations (9). Thus, distribution of water, which constitutes the bulk of ISF and CSF, is very limited due to its rapid turnover across the microvascular walls. It is important to emphasize that microcirculatory/microvessel hypothesis interconnects the physiology of all craniospinal fluids (plasma, intracellular, extracellular, and cerebrospinal fluid). This new concept of CSF and other fluids dynamics opens many possibilities in the investigation of severe clinical problems such as normal pressure, arrested oracute hypertensive hydrocephalus, intracranial hypertension, and focal and generalized brain edema.

This issue of the *Croatian Medical Journal* (CMJ) is dedicated to the CSF dynamics, and we hope that it will provide the readers with new data and views that can help them in their research projects. Due to the significance of the new CSF dynamics concept, this issue’s editorial is supplemented by a reprint of the article by Bulat and Klarica published in the *Periodicum Biologorum* in 2005, which first presented the new hypothesis on fluids physiology (11) (Supplementary material(supplementary material)). This issue also offers several comprehensive reviews on different aspects of CSF functioning. Gato et al describe embryonic CSF before the development of the choroid plexuses and show different novel concepts of embryonic CSF (eCSF) functioning, while Bueno et al in their extensive and detailed review of a very early protective barrier (embryonic blood-CSF barrier) explain the control of internal milieu (components of eCSF) in a developing brain. Orešković and Klarica describe methodological errors of traditional and most widely accepted perfusion method for measuring CSF formation and absorption. The article by Nakada discusses a new concept of CSF physiology, describing the importance of Virchow-Robin space and aquaporin 4 in the functioning of CNS, while that by Yamada describes novel aspects of CSF physiological and pathophysiological movement visualized by a new non-invasive MRI technique (Time-SLIP). Babić et al discuss the current status of a great variety of CSF biomarkers for the use in Alzheimer disease diagnostics and Krishnamurthy et al summarize the data about pathophysiological mechanisms of hydrocephalus development induced by an application of hyperosmolar solution into the CSF space, and hypothesize that impaired efflux at the blood-brain barrier will result in an increased concentration of different substances inside the brain interstitial and ventricular fluid, leading to hydrocephalus development.

This issue also presents reports on several cases that can in no way be explained by the generally accepted traditional hypothesis. Such is the case of idiopathic CSF “hypersecretion” (Trevisi et al) in a 6-month-old infant, which cannot be controlled using standard operative drainage techniques (the article also gives an overview of the available data regarding CSF formation). There is also the case of the oldest living patient with hydranencephaly, which raises some questions regarding the CSF turnover and homeostasis in a person with no brain parenchyma inside the supratentorial space (Radoš et al). Also, this issue includes a case of a severe aqueductal stenosis lasting for 5 years without any detectable CSF movements, which is not accompanied with hydrocephalus development (Radoš et al). In addition, Bechter and Shmitz describe time dynamics of contrast distribution from lumbar subarachnoid space into the psoas muscle tissue in one patient, and discuss the importance of their findings for pain research.

## Marin Bulat's contribution to the physiology and pathophysiology of the cerebrospinal fluid

**Figure 1:**
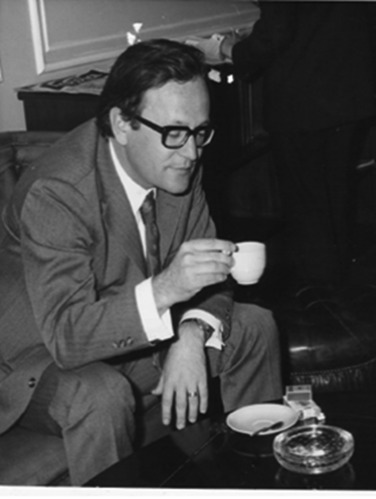
Professor emeritus Marin Bulat (1936-2012)

**Figure 2:**
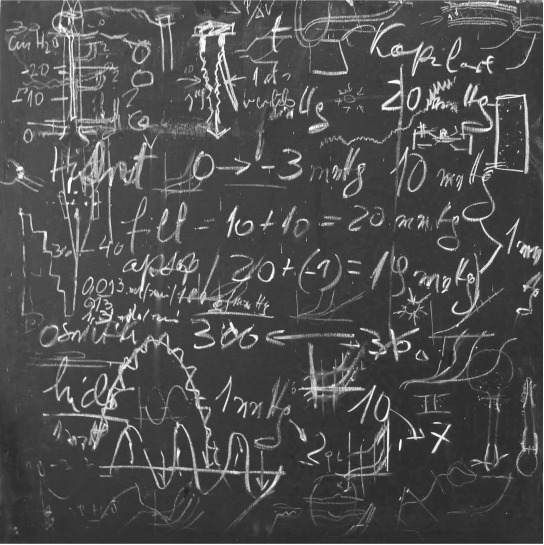
A blackboard in Prof. Bulat's study after many hours of discussion about the dynamics of cerebrospinal fluid and other intracranial fluids.

Due to the importance of his reserch for disputing the traditional CSF physiology hypothesis, we dedicate a part of this editorial to the work of Prof. emeritus Marin Bulat (1936-2012), who started the CSF physiology research in Croatia (Figure 1, Figure 2).

Marin Bulat began his scientific career at the Ruđer Bošković Institute under the mentorship of Prof. Zlatko Supek. In his master's (1964) and PhD thesis (1966), which determined the course of his scientific work, he explored the role of serotonin and its metabolites inside the CSF system and the brain. His early papers describe the serotonin passage from CSF into the brain by means of diffusion process (without any restrictions) depending on the concentration gradient (12,13). Serotonin is very quickly metabolized after its passage from CSF into the brain tissue and its metabolite 5-HIAA quickly disappears from the site of its formation. He showed that serotonin did not distribute inside the CSF and that its lumbar CSF concentration merely reflected the changes inside the surrounding spinal cord tissue (14,15). Namely, the 5-HIAA concentration changes after its application into the cisterna magna did not influence its concentration inside the lumbar subarachnoid space. However, based on the classic hypothesis of CSF physiology, increased concentration inside the lumbar subarachnoid space would be reasonably expected. As he noticed that an increase in metabolic transformation inside the spinal cord tissue led to an increase in the concentration inside the surrounding CSF, he concluded that the changes of 5-HIAA concentration inside the lumbar CSF reflected only the local metabolism inside the spinal cord tissue (14,16).

On the basis of these observations, an idea developed that substances were not distributed throughout the CSF according to the classic hypothesis of CSF physiology. When molecules with different molecular weight were monitored, such as radioactive water, organic acids (5-HIAA, ^3^H - benzylpenicillin, phenolsulphonphtalein) or inulin inside the CSF system and CNS, it was observed that they were distributed in all directions and that the distribution intensity depended on the rate of their elimination into the CNS capillaries (9,17,18). According to the generally accepted hypothesis, the CSF is secreted inside the brain ventricles and flows unidirectionally along the subarachnoid spaces to be absorbed into the dural venous sinuses. However, a small molecule like water, which constitutes 99% of CSF bulk, does not flow unidirectionally along the CSF spaces since it is rapidly absorbed into the adjacent microvessels (19).

The distribution of substances with long residence time (inulin, proteins, etc) caused by to-and fro pulsations will always, after their application into the lateral ventricles (LV), create an illusion of a unidirectional bulk CSF circulation (from LV to cisterna magna, cortical and spinal subarachnoid space). On the contrary, after application of these substances into other parts of the CSF system, they are distributed in all directions (as well as into the LV, which is contradictory to the classical hypothesis) (9). Besides this, it can be observed that the arrival of inulin into the area of lumbar CSF is much faster than into the cortical subarachnoid space. All of this implies that the CSF moves much differently than what was previously concieved. After a series of experiments on the influence of CSF and blood hydrostatic and osmotic pressure on the CSF volume and pressure had been performed, a new hypothesis was suggested, according to which interstitial fluid and CSF make a single functional unit, while CNS microvessels are crucial for the fluid absorption and filtration (9).

In his fight against the conventional notions, Prof. Bulat found inspiration and strenght in the thought of Claude Bernard, one of the most distinguished physiologists and medical scientists of the nineteenth century: ”When we meet a fact which contradicts a prevailing theory, we must accept the fact and abandon the theory, even when the theory is supported by great names and generally accepted.” He never gave up, and he managed to show his students and colleagues the way to persevere in science. He used to say that science is like a candle that burns low, and that this flame should be preserved and carried on with extreme attention, scientific integrity, and hard persistent work. He passed on this difficult task to all of us who are now, together with our foreign colleagues, trying to save that flame of science and to pass it on to new generations.

Instead of conclusion let's thank Prof. emeritus Marin Bulat with another famous sentence by Claude Bernard: “A man of science rises ever, in seeking truth; and if he never finds it in its wholeness, he discovers nevertheless very significant fragments; and these fragments of universal truth are precisely what constitutes science.”
