# Supplementary material for: Enigma of cerebrospinal fluid dynamics
Source: Croat Med J. 2014 Aug;55(4):287–98. doi: 10.3325/cmj.2014.55.287 (PMC4157379; doi:10.3325/cmj.2014.55.287)
Supplement: Supplementary material [file CroatMedJ_55_s011.pdf]

Fluid filtration and reabsorption across microvascular walls: control by oncotic or osmotic pressure? (secondary publication)

Marin Bulat and Marijan Klarica

Department of Pharmacology and Croatian Institute for Brain Research,University of Zagreb School of Medicine, Zagreb, Croatia

The article represents a secondary publication identical to previously published paper Bulat M, Klarica M. Fluid filtration and reabsorption across microvascular walls: control by oncotic or osmotic pressure? Period Biol. 2005;107:147-52. Published with premission from Periodicum biologorum.

Aim. Relationships between hydrostatic and oncotic (colloid osmotic) pressures in both capillaries and interstitium are used to explain fluid filtration and reabsorption across microvascular walls. These pressures are incorporated in the Starling oncotic hypothesis of capillaries which fails, however, to explain fluid homeostasis when hydrostatic capillary pressure is high (in feet during orthostasis) and low (in lungs), or when oncotic plasma pressure is significantly decreased in experiments and some clinical states such as genetic analbuminaemia.

Methods. To explain fluid homeostasis we propose osmotic counterpressure hypothesis of capillaries which claims: 1) during water filtration across microvascular wall in arterial capillary, the plasma osmolytes are sieved (retained) so that plasma osmotic counterpressure is generated, 2) this osmotic counterpressure rises along the length of capillary and when it reaches capillary hydrostatic pressure the water filtration is halted, and 3) in venous capillaries and postcapillary venules where hydrostatic pressure is low, the osmotic counterpressure is instrumental in water reabsorption from interstitium what leads to dissipation of osmotic counterpressure. According to modified van't Hoff's equation the generation of osmotic counterpressure depends on plasma concentration of osmolytes and their restricted passage (reflection coefficient) across microvascular wall in comparison to water.

Results. Plasma NaCl makes 83% of plasma osmolarity and shows restricted passage across the walls of cerebral and peripheral continuous capillaries, so that Na and Cl are the most important osmolytes for generation of osmotic counterpressure. Our calculation indicates that at various rates of water filtration the osmotic counterpressure of NaCl acts as negative feedback control: higher hydrostatic pressure and water filtration rate create higher osmotic counterpressure which opposes filtration and leads to higher water reabsorption rate. Furthermore, our analysis indicates that fluid volume changes in arterial capillaries are proportionally 100 times larger than in interstial fluid.

Conclusion. The osmotic counterpressure hypothesis explains fluid homeostasis at high, mean and low capillary hydrostatic pressures. Plasma proteins and inorganic electrolytes contribute 0.4% and 94% to plasma osmolarity, respectively, so that plasma proteins have low osmotic (oncotic) pressure and despite high restriction of their passage across microvascular wall they contribute little to build up of osmotic counterpressure in comparison to electrolytes. However, absence or very low concentration of plasma proteins increases microvascular wall permeability to water and osmolytes compromising build up of osmotic counterpressure leading to development of interstial oedema.

Correspondence:

Marijan Klarica MD, PhD
Department of Pharmacology
University of Zagreb School of Medicine
Šalata 11, HR - 10 000 Zagreb, Croatia.
Tel.: + 385 – 1 – 45 – 66 – 839
Fax: + 385 – 1 – 45 – 96 – 942
E-mail: mklarica@hiim.hr

INTRODUCTION

Microvascular vessels include arterial and venous capillaries and postcapillary venules where filtration and reabsorption of water volume and exchange of solutes take place. Microvascular walls are made of single layer of flattened endothelial cells and intercellular junctions which are covered intraluminally by a negatively charged coat called glycocalyx (1,2). In the development of concept of fluid filtration and reabsorption it was assumed that water and all plasma solutes except proteins pass freely across microvascular walls. This concept is known as Starling hypothesis of capillaries named in honour of E.H. Starling who first proposed it in 1896 (3). The hypothesis was elaborated in 1963 by Landis and Pappenheimer (4), and with small modifications it represents an important chapter in contemporary textbooks of physiology. The Starling hypothesis is used to explain maintenance of fluid homeostasis in body and development of fluid disbalance in various pathological conditions leading to interstial oedema.

The Starling hypothesis of microvessels claims that rate of fluid filtration and reabsorption of fluid volume (J_v, volume/time) across microvascular walls is regulated by hydrostatic pressure in the capillary (HP_c) and interstitium (HP_i) and oncotic or colloid osmotic pressure of proteins in the capillary (COP_c) and interstitium (COP_i) according to the equation [4]:

$$J_v = L_p\,[(HP_c - HP_i) - (COP_c - COP_i)] \quad [1]$$

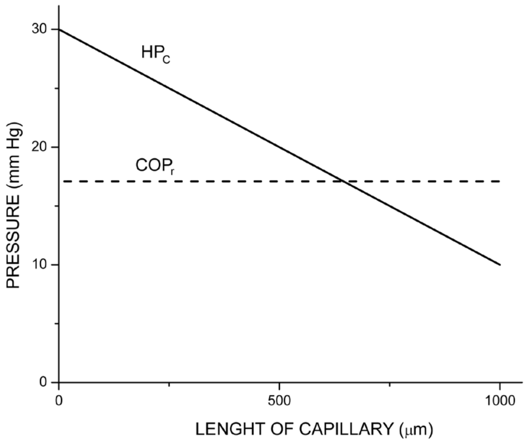

FIGURE 1. Schematic presentation of the Starling oncotic hypothesis of capillaries. HP_c, hydrostatic capillary pressure; COP_r, reabsortive oncotic pressure. Fluid filtration takes place when $HP_c > COP_r$, while fluid reapsorption occurs when $HP_c < COP_r$. For explanation see text.

where L_p is hydraulic conductivity of the microvascular wall. Positive J_v means fluid movement out of capillary (filtration), negative into capillary (reabsorption). The effects of the Starling pressures on fluid filtration and reabsorption are shown in a simplified way in Fig. 1 along capillary of 1000 µm length. HP_c is 30 mmHg at the arterial end but due to resistance to blood flow it falls to 10 mmHg at the venous end of the capillary. HP_c tends to force fluid outwards through the capillary wall. HP_i is omitted in Fig. 1 since it is close to zero, i.e. few mmHg positive or negative (subatmospheric) in most tissues. COP_c is 25 mmHg and it tends to cause reabsorption of fluid from interstitium which is opposed by COP_i of 8 mmHg. Thus, when COP_i (8 mmHg) is subtracted from COP_c (25 mmHg) the reabsorptive oncotic pressure (COP_r) of 17 mmHg is obtained (Fig. 1). According to the Starling oncotic hypothesis the filtration of fluid takes place in arterial part of the capillary where $HP_c > COP_r$, whereas reabsorption of fluid occurs in venous capillary and postcapillary venules where $COP_r > HP_c$. Furthermore, it is assumed that COP_r does not change significantly since volumes of filtered and reabsorbed fluid are relatively small, and that a significant part of filtered fluid is absorbed in the lymphatic capillaries.

There are some data, however, which indicate that COP_r may not be a decisive factor in regulation of fluid filtration and reabsorption. In the development of the Starling oncotic hypothesis it was assumed that only plasma proteins show restricted passage across capillary walls, while all other plasma solutes pass relatively freely and cannot exert a significant osmotic pressure between plasma and interstitium. However, it is known that cerebral capillaries are negligibly permeable to inorganic ions (5,6) and that peripheral continuous capillaries restrict significantly passage of these solutes (7,8), what should be incorporated into any comprehensive hypothesis of capillaries. The values of HP_c in Fig. 1 are used arbitrary at heart level but these values may be much higher or lower so that COP_r might not be able to control fluid filtration and reabsorption. In humans in upright position the HP_c in feet nailfolds is above 90 mmHg so that COP_r by itself could not prevent fast development of feet oedema (2,9). In the lung capillaries the HP_c is about 7 mmHg, i.e. much lower than COP_r of 17 mmHg, and it is not clear how filtration of fluid can take place (10). When plasma proteins were decreased 65% by plasmapheresis in rabbits, no brain water increase was observed indicating that the Starling oncotic hypothesis is not operative in cerebral capillaries (11). In addition, in patients with genetic analbuminaemia the COP_c is decreased by 50% without development

of oedema what is difficult to explain by the Starling hypothesis (4,12).

In attempt to resolve these problems of the Starling oncotic hypothesis, we propose the osmotic counterpressure hypothesis of the capillaries which claims that not COP_r but plasma osmotic pressure changes in the capillaries (OP_c) are instrumental in regulation of water filtration and reabsorption. However, plasma proteins are important for maintenance of normal permeability of microvascular walls to water and solutes (4,13,14), but they contribute little to plasma effective osmotic pressure (see below).

ASSUMPTIONS OF THE OSMOTIC COUNTERPRESSURE HYPOTHESIS

Osmolarity is defined as concentration of osmotically active particles (osmolytes), and is usually expressed in milliosmoles per litre of water (mosm/l). Plasma and interstial fluid osmolarities are about 300 mosm/l (15), excluding kidney as special organ which is not considered here. Plasma Na and Cl constitute 142 and 108 mosm/l, respectively (15), what makes 83% of plasma osmolarity, while contribution of other inorganic ions (HCO_3, K, Ca, Mg, HPO_4, H_2PO_4, SO_4) is 11%. Thus, while inorganic ions contribute 94% to plasma osmolarity, contribution of proteins and other organic substances (glucose, urea, aminoacids, lactate, creatine) is only 0.4% and 4.3 %, respectively. Since all mentioned plasma osmolytes are hydrophilic substances their passage across cell membrane and microvascular wall may be restricted, while plasma lipophilic substances (e.g. CO_2 and O_2) diffuse easily through membranes and do not contribute to effective osmotic pressure.

Difference of effective osmotic pressure (ΔOP) between two compartments separated by membrane or microvascular wall is calculated according to the modified van't Hoff equation (16):

$$\Delta OP\ (mmHg) = \Delta C_{mosm}\ \sigma\ R\ T \quad [2]$$

where ΔC_{mosm} is concentration difference of osmolytes (mosm/l), σ is reflection coefficient of osmolytes, T is absolute temperature (ºK) and R is universal gas constant (0.06236 mmHg per mosm/l and degree ºK). At normal body temperature of 37 ºC (310 ºK), RT (0.06236 x 310) equals 19.3 mmHg per mosm/l (16). Reflection coefficients (σ) of osmolytes indicate how they are «reflected» from microvascular walls during water passage under hydrostatic or osmotic pressure, and theoretically they may range from 1 representing complete impermeability (100% «reflection») down to 0, for a solute permeability equal to that of water (σ = 0) (2). As discussed below, the inorganic ions such as Na and Cl have σ significantly higher than 0 (water), so that they should affect filtration and reabsorption of water. In consideration of transcapillary movement of water and osmolytes it is usually assumed that hydrostatic pressure drives water through specific water-only pathway, while water and small osmolytes are driven through small pores and proteins through a few large pores.

Equation [2] indicates that osmotic pressure (OP) of osmolytes in linearly related to their concentration, what is not the case for plasma proteins. For calculation of COP of plasma proteins Landis and Pappenheimer developed an empirical equation (4):

$$COP\ (mmHg) = 2.1\ c + 0.16\ c^2 + 0.009\ c^3 \quad [3]$$

where c is concentration of proteins expressed in grams per 100 ml of plasma. As fluid begins to be filtered through wall of arterial capillary, its composition is determined by the rates at which different plasma osmolytes can move by convention or diffusion in comparison to water. Concentration of a plasma osmolyte with σ > 0 should increase during water (σ = 0) filtration in arterial capillary since proportionally more water than osmolyte should pass across capillary wall (17). In such a way concentration of osmolyte should increase in arterial capillary creating an osmotic counterpressure (OcP_c) which opposes the water filtration. Thus, OcP_c is osmotic pressure increase in arterial capillary above normal OP present in systemic blood circulation. According to Equation [2] this OcP_c depends on plasma concentration and σ of the osmolyte, as well as on water filtration rate. The estimation of water filtration rates in peripheral continuous capillaries are 1 - 4% of the plasma volume flow depending on HP_c (1,4), while this rate should be lower in cerebral capillaries (6). Taking a range of capillary water filtration rates and known σ of plasma osmolytes, the capillary osmotic counterpressure (OcP_c) opposing water filtration can be calculated (see below).

OSMOTIC COUNTERPRESSURE IN CEREBRAL CAPILLARIES

Cerebral capillaries form the blood-brain barrier and are characterized by endothelial cells with tight intercellular junctions which encircle completely each endothelial cell. Water permeability of cerebral capillaries is relatively high (18), while the passage of proteins and

electrolytes is very limited (6), so that reflection coefficient (σ) of proteins is 0.999 (1), and σ of Na and Cl about 0.98 could be estimated (6, 19, 20). If we take plasma concentration of Na 142 mosm/l (see above), at 0.2% water filtration rate this number of milliosmols would be contained in 0.998 l of plasma due to water loss. When concentration of Na is recalculated per l of plasma, we obtain (142 mosm/ 0.998 l) 142.285 mosm/l or an increase of 0.285 mosm/l. Similar calculation for Cl shows that its normal concentration of 108 mosm/l would rise to 108.216 mosm/l, or an increase of 0.216 mosm/l. Thus, total increase of Na and Cl osmolarity (0.285 + 0.216) is 0.501 mosm/l. For calculation of osmotic pressure this value should be multiplied by osmotic coefficient for NaCl which is 0.93 (16), so we obtain (0.501 x 0.93) 0.466 mosm/l. Taking σ = 0.98 for NaCl (see above) calculated osmotic counterpressure in cerebral capillaries (OcP_c) according to the Equation [2] is: 0.466 x 0.98 x 19.3 = 8.81 mmHg.

Thus, at water filtration rate of 0.2% an OcP_c of NaCl about 9 mmHg is generated in arterial capillaries. The rate of water filtration can increase or decrease depending on changes of HP_c. In Table 1. are shown some values of OcP_c of NaCl at different rates of water filtration. These values of OcP_c are not permitted to run down by diffusion and/or convention of NaCl across capillary wall since they are continuously maintained by plasma flow and fluid filtration.

It can be calculated that no significant oncotic counterpressure in plasma is generated during 0.2% water filtration rate. If concentration of plasma proteins is 7 g/100 ml, than at water filtration rate of 0.2%, this concentration would increase to 7.014 g/100 ml due to water loss. When these concentrations of proteins are included in Equation [3], the calculated COP_c are 25.627 and 25.706 mmHg, respectively. Thus, calculated oncotic counterpressure of plasma proteins (25.706 – 25.627) is 0.079 mmHg which does not change when multiplied by σ for proteins which is 0.999 (see above). Such a small OcP_c of plasma proteins is not physiologically significant.

HP_c in cerebral capillaries is not known, but hydrostatic pressure in pial arterioles (25 μm d.) penetrating in brain parenchyma is 55 mmHg in cats (*21*), while pressure in pial venules (100 – 200 μm d.) leaving parenchyma is 4 mmHg in rats (22). This suggests that a relatively high axial gradient of hydrostatic pressure along microvascular bed is present, where filtration and reabsorption of water occur. Since HP_c falls (Fig. 1) and OcP_c of NaCl rises along length of the capillary due to filtration of water and retention (sieving) of NaCl, at a point these pressures should become equal so that filtration equilibrium is reached, i.e. water filtration is brought to halt (Fig. 2). When such hypertonic plasma is delivered to venous capillaries and postcapillary venules, where HP_c is lower than OcP_c, osmotic reabsorption of water from interstial fluid into these vessels takes place (Fig. 2). Due to water reabsorption the hypertonic plasma is diluted and finally normalized in postcapillary venules (not shown in Fig. 2), i.e. OcP_c is dissipated. Thus, according to our osmotic counterpressure hypothesis normal osmolarity in systemic blood circulation changes in microvessels: increase of osmolarity (OcP_c) is generated in arterial capillaries due to water filltration and NaCl sieving (retention), while in venous capillaries and postcapillary venules this increased osmolarity (OcP_c) is dissipated due to water reabsorption so that normal plasma osmolarity is delivered to veins. In the other words, when $HP_c > OcP_c$ filtration of wa-

TABLE 1. Calculated osmotic counterpressures (OcP_c) of NaCl in cerebral and skeletal muscle capillaries (mmHg) at different water filtration rates expressed in percentages (%) of plasma volume flow.

Cerebral capillaries		Skeletal muscle capillaries	
Filtration rate	OcP_c of NaCl	Filtration rate	OcP_c of NaCl
0.10%	4.40 mmHg	0.25%	5.63 mmHg
0.20%	8.81 mmHg	0.50%	11.28 mmHg
0.40%	17.66 mmHg	1.00%	22.66 mmHg
0.80%	35.46 mmHg	2.00%	45.79 mmHg
1.00%	44.41 mmHg	4.00%	93.49 mmHg

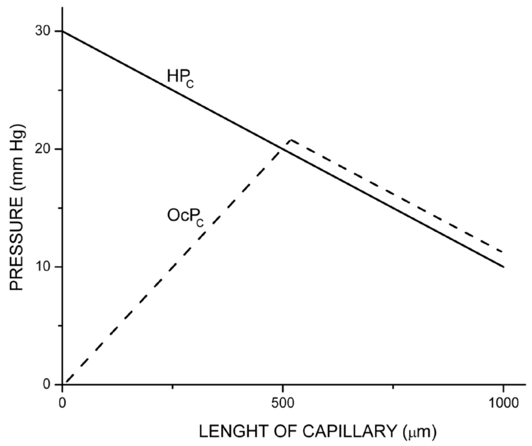

FIGURE 2. Schematic presentation of osmotic counterpressure hypothesis of capillaries. HP_c, hydrostatic capillary pressure; OcP_c, capillary osmotic counterpressure. Fluid filtration takes place when $HP_c > OcP_c$, while fluid reapsorption occurs when $HP_c < OcP_c$. For explanation see text.

ter takes place, whereas when $OcP_c > HP_c$ reabsorption of water occurs leading to isosmolar plasma.

To keep this analysis simple we omit here the contribution of other plasma osmolytes except NaCl to development of OcP_c during water filtration. Namely, other plasma inorganic and organic osmolytes (see above) should contribute to development of OcP_c since they show limited capillary permeability (5,6). Various transport processes across microvascular walls such as facilitated influx of D-glucose (1) and aminoacids (23) or active efflux of organic acids (24,25,26), as well as transport of some inorganic ions (27), could contribute to a long-term maintenance of osmotic homeostasis in the brain. We assume that water filtration does not change significantly volume and osmolarity of interstial fluid because cerebral capillaries form a dense and interconnected networks of vessels so that simultaneous filtration and reabsorption of water takes place everywhere between numerous adjacent capillary branches preventing significant changes of volume and osmolarity in interstitium (see below). It should be mentioned that no lymphatic system is present in brain so that only blood microvessels are instrumental in water and solutes reabsorption.

OSMOTIC COUNTERPRESSURE IN PERIPHERAL CAPILLARIES

The most abundant peripheral capillaries are those with continuous uninterrupted endothelial cells connected by intercellular junctions. These capillaries are found in skeletal, smooth and cardiac muscles, skin, lungs and connective tissues (1). Some studies gave somewhat different permeabilities of continuous capillaries for inorganic ions although it seems probable that these permeabilities in various organs are similar (28). In determination of σ for various osmolytes in microvessels two factors seems to be especially important: composition of perfusate and rate of perfusion. When capillaries are perfused with protein-free perfusate an increase in hydraulic conductivity and osmolytes permeability of microvascular walls is observed indicating that this structure lose its selectivity (4,13,14). In addition, if rate of perfusion is not sufficiently higher than permeability-surface product of microvessels for the solute studied, its reflection coefficient is underestimated (7,8).

In skeletal and heart muscle σ of NaCl is 0.50 or higher (7,8). If water filtration rate in skeletal muscles is 1% of plasma volume flow, normal Na plasma concentration of 142 mosm/l should increase to 143.434 mosm/l and Cl from 108 mosm/l to 109.091 mosm/l, respectively, due to water loss. Thus, total increase of Na and Cl (1.434 + 1.091) is 2.525 mosm/l. When this value is multiplied by osmotic coefficient for NaCl which is 0.93, we obtain 2.348 mosm/l. Taking σ for NaCl 0.50, the OcP_c for NaCl according Equation [2] is: 2.348 x 0.50 x 19.3 = 22.66 mmHg.

Thus, at 1% water filtration rate the OcP_c of NaCl of about 23 mmHg is generated. In Table 1 is shown OcP_c at different water filtration rates caused by different HP_c. When OcP_c of NaCl reaches the same value as HP_c along the length of arterial capillaries the water filtration should be halted, while in venous capillaries and postcapillary venules the OcP_c should assist in water reabsorption from interstitium as suggested above for cerebral capillaries (Fig. 2). When water filtration rate in skeletal muscle capillaries is 1%, concentration of plasma proteins 7 g/100 ml and σ of plasma proteins 0.90 (*1*), the calculated oncotic counterpressure (see above) is 0.4 mmHg, a very small contribution to OcP_c of NaCl.

As already mentioned HP_c in feet capillaries of humans in upright position is somewhat above 90 mmHg (9). Assuming 4% water filtration rate in such a case, an OcP_c of above 90 mmHg would be generated as can be seen in skeletal muscle capillaries in Table 1, what should prevent medically relevant development of feet oedema. On the contrary, the HP_c in pulmonary capillaries is low, about 7 mmHg (10), indicating that both rate of water filtration and generated OcP_c should be also low. However, pulmonary capillaries have great density so that rate of water filtration and reabsorption could be considerable per gram of tissue. Furthermore, we assume that intermittent changes of HP_c as occur in vasomotion and pulse pressure should contribute to fine tuning of fluid filtration and OcP_c according to metabolic needs of tissues. Since in peripheral tissues the lymphatic system is present a part of filtered fluid is absorbed in lymphatic capillaries and returned to bloodstream by lymph flow.

To get an insight in osmotic power of total plasma OP_c in comparison to COP_c of plasma proteins we can calculate their effective osmotic pressures which would develop across microvascular wall assuming that interstial fluid is pure water. Taking plasma osmolarity 300 mosm/l and σ = 0.50 for all plasma osmolytes, the effective OP_c would be according to Equation [2]: 300 x 0.50 x 19.3 = 2895 mmHg, or 116 times higher than COP_c of plasma proteins which is 25 mmHg. If we take σ = 0.10 for all plasma osmolytes, the effective OP_c would be 579 mmHg, or 23 times greater than COP_c. This analysis indicates that

osmotic power of total plasma osmolarity is much greater than COP_c, and that OcP_c, but not COP_c or COP_i, should control fluid filtration and reabsorption across microvascular walls as elaborated above.

Plasma proteins and some other blood components are important for maintenance of integrity and normal permeability of microvascular walls. When microvessels are perfused with protein-free or blood-free perfusate the hydraulic conductivity and permeability to plasma solutes of microvascular walls increases several times (13,14). Under such conditions it is expected that σ and OcP_c of NaCl and other plasma osmolytes should decrease what would compromise normal filtration and reabsorption of fluid across microvascular wall and lead to development of interstial oedema.

Our hypothesis of role of NaCl in regulation of water volume passage across microvascular walls is supported by some experimental and clinical observations. When hyperosmolar NaCl solution is applied intravascularly, the absorption of water from interstitium in skeletal muscles is greatly increased (29). In addition, hyperosmolar NaCl solution applied intravascularly in patients with increased intracranial pressure leads to augmented water reapsorption from brain parenchyma and fall of increased intracranial pressure (30,31). Restricted passage of NaCl in comparison to water across microvascular walls, as suggested by our osmotic counterpressure hypothesis, explains these observations.

Fenestrated capillaries are present in some tissues such as exocrine and endocrine glands, gastrointestinal mucosa and kidney (1). These vessels are characterized by circular fenestrae or pores that penetrate the endothelium which are usually closed by a very thin diaphragm. Fenestrated capillaries show high permeability to water and inorganic ions (32). Since we were not able to find published data of σ of inorganic ions in those vessels it is impossible to guess at this time whether high water filtration rate could lead to such increase of their osmolarity and OcP_c which would reach the filtration equilibrium. However, such a possibility should not be a priori excluded.

CAPILLARY OSMOTIC COUNTERPRESSURE AS NEGATIVE FEEDBACK CONTROL

Our osmotic hypothesis indicates that the OcP_c acts as negative feedback control which opposes the water filtration: higher HP_c and water filtration rate create higher OcP_c (Table 1), which halts water filtration (Fig. 2). Furthermore, this OcP_c in venous capillaries and postcapillary venules is instrumental in water reabsorption what dissipates OcP_c. Thus, the water filtration and reabsorption rate (J_v) can be expressed by following relation:

$$J_v = L_p\,(\Delta HP_{c-i} - \Sigma OcP_c) \quad [4]$$

where ΔHP_{c-i} is difference of HP_c and HP_i, and ΣOcP_c is sum of counterpressures of all plasma osmolytes with σ > 0. As already discussed this ΣOcP_c is mostly due to NaCl and other inorganic ions which constitute 94% of total plasma osmolarity.

The question arises how a sudden increase of HP_c and water filtration rate from 1% to 4% would be reflected in interstial fluid volume. In man volumes of blood and plasma are 5 l and 3 l, respectively, volume of blood in the capillaries is 4% of total blood volume (0.20 l of blood and 0.12 l of plasma) (10,33) while volume of interstial fluid is 12 l. When 1% of capillary plasma volume (0.0012 l) is filtered into 12 l of interstial fluid, the interstial fluid volume would increase by 0.01%, while at 4% filtration rate (0.0048 l) this increase would be 0.04%. Thus, volume fluid changes in the arterial capillaries are proportionally 100 times or two order of magnitude larger than those in interstial fluid. Since filtration and reabsorption of fluid are simultaneous processes, such minute increases of interstial fluid volumes should be easily compensated by fluid absorption. Due to such minute changes of interstial fluid volume we assume that osmolarity and pressure of interstial fluid change very little in comparison to such changes in microvessels.

In conclusion, our osmotic counterpressure hypothesis of the capillaries suggests that osmotic counterpressure of plasma osmolytes is the main regulator of water filtration and reabsorption across microvascular walls and principal controller of interstial fluid volume in physiological conditions. However, when permeability of microvascular walls is increased due to various pathological processes including significant hypoproteinaemia, the reflection coefficient of plasma osmolytes and their osmotic counterpressure should decrease while hydraulic conductivity of microvascular should increase leading to development of interstial oedema.

Acknowledgments This work wasupported by the Croatian Ministry of Science and Technology.

Competing interests: The authors have declared that no competing interest exists.

Abbreviations

COP_c – capillary colloid osmotic (oncotic) pressure

COP_i – interstitial oncotic pressure

COP_r – reabsorptive oncotic pressure

HP_c – capillary hydrostatic pressure

HP_i – interstitial hydrostatic pressure

Jv – rate of fluid filtration or reabsorption

Lp – hydraulic conductivity of capillary wall

OcP_c – capillary osmotic counterpressure

OP_c – capillary osmotic pressure

References

1 Renkin E M, Crone C. Microcirculation and capillary exchange. In: Greger R, Windhorst U, editors. Comprehensive human physiology. Berlin: Springer; 1996. p. 1965-79.

2 Michel CC. Exchange of fluid and solutes across microvascular walls. In: Seldini D W, Giebisch G, editors. The kidney. 3rd ed. Philadelphia: Lippincott Williams & Wilkins; 2000. p 61-84.

3 Starling EH. On the absorption of fluids from connective tissue spaces. J Physiol (Lond). 1896; 19: 312-26.

4 Landis EM, Pappenheimer JR. Exchange of substances through the capillary walls. In: Hamilton W F, Dow P, editors. Handbook of physiology, Circulation Vol. II. Washington DC: American Physiological Society; 1963. p. 961-1034.

5 Katzman R, Pappius HM. Brain electrolytes and fluid metabolism. Baltimore: Williams & Wilkins; 1973.

6 Fenstermacher JD, Rapoport SI. Blood-brain barrier. In: Renkin EM, Michel CC, editors. Handbook of physiology, Microcirculation, Vol. IV. Bethesda MD: American Physiological Society; 1984. p. 969-1000.

7 Yudilevich DL, Alvarez OA. Water, sodium and thiourea transcapillary diffusion in the dog heart. Am J Physiol. 1967; 213: 308-314.

8 Wolf MB, Watson PD. Measurement of osmotic reflection coefficient for small molecules in cat hindlimbs. Am J Physiol. 1989; 256: H282 – H290.

9 Levick JR, Michel CC. The effects of position and skin temperature on the capillary pressures in the fingers and toes. J Physiol (Lond). 1979; 274: 97-109.

10 Staub NC, Dawson CA. Pulmonary and bronchial circulation. In: Greger R, Windhorst U, editors. Comprehensive human physiology. Berlin: Springer; 1996. p. 2071-2078.

11 Zornow MH, Todd MM, More SS. The acute effects of changes in plasma osmolarity and oncotic pressure. Anesthesiology 1987; 67: 936-941.

12 Benhold H, Klaus D, Scheurlen PG. Volume regulation and renal function in analbuminaemia. Lancet 1960; 2: 1169 – 1170.

13 Watson PD. Effects of blood-free and protein-free perfusion on CFC in the isolated cat hindlimb. Am J Physiol. 1983; 245: H911-H919.

14 Huxley VH, Curry FE. Differential actions of albumin and plasma on capillary solute permeability. Am J Physiol. 1991; 260: H1645-H1654.

15 Guyton AC, Hall JE. Textbook of medical physiology. 9th ed. Philadelphia: WB Saunders; 1996. p. 301.

16 Baumgarten CM, Feher JJ. Osmosis and regulation of cell volume. In: Sperelakis N, editor. Cell physiology sourcebook. Academic Press, San Diego, 2001. p. 319-355.

17 Bulat M, Klarica M. Osmotic control of intracranial pressure. Period Biol. 2001; 103: 293-296.

18 Bolwing TG, Lassen NA. The diffusion permeability to water of the rat blood-brain barrier. Acta Physiol Scand. 1975; 93: 415-422.

19 Yudilevich DL, De Rose N. Blood-brain transfer of glucose and other molecules measured by rapid indicator dilution. Am J Physiol. 1971; 220: 841-846.

20 Fraser PA, Dallas AD. Measurement of filtration coefficient in single cerebral microvessels of the frog. J Physiol (Lond.). 1990; 423: 343-361.

21 Shapiro HM, Stomberg DD, Lee DR, Wiederhielm CA. Dynamic pressures in the pial arterial microcirculation. Am J Physiol. 1971; 221: 279-283.

22 Wiig H, Reed RK. Rat brain interstial fluid pressure measured with micropipettes. Am J Physiol. 1983; 244: H239-H246.

23 Smith QR, Stoll J. Blood-brain barrier amino acid transport. In: Pardrige WM, editor. Introduction to the blood-brain barrier. Cambridge: Cambridge University Press; 1998. p. 188-197.

24 Bulat M, Živković B. Exchange of 5-hydroxyindoleacetic acid between spinal cord and lumbar cerebrospinal fluid. J Physiol (Lond). 1978; 275: 191-197.

25 Vladić A, Strikić N, Jurčić D, Zmajević M, Klarica M, Bulat M. Homeostatic role of the active transport in elimination of 3H-benzylpenicillin out of the cerebrospinal fluid system. Life Sci. 2000; 67: 2375-2385.

26 Zmajević M, Klarica M, Varda R, Kudelić N, Bulat M. Elimination of phenolsulfonphthalein from the cerebrospinal fluid via capillaries in the central nervous system in cats by active transport. Neurosci Lett. 2002; 321: 123-125.

27 Keep RF, Ennis SR, Betz AL. Blood-brain barrier ion transport. In: Pardridge WM, editor. Introduction to the blood-brain barrier. Cambridge: Cambridge University Press; 1998. p. 207-213.

28 Crone C, Levitt DG. Capillary permeability to small solutes. In: Renkin EM, Michel CC, editors. Handbook of physiology. Microcirculation Vol. IV. Bethesda MD: American Physiological Society; 1984. P. 441-466.

29 Hamilton MT, Ward DS, Watson PD. Effect of plasma osmolarity on steady-state fluid shifts in perfused cat skeletal muscle. Am J Physiol. 1993; 265: R1318-R1323.

30 Saltarini M, Massarutti D, Baldassarre M, Nardi G, de Cole C, Fabris G. Determination of cerebral water content by magnetic resonance imaging after small volume infusion of 18% hypertonic saline solution in a patient with refractory intracranial hypertension. Eur J

Emerg Med. 2002; 9: 262-265.

31 Doyle JA, Davis DP, Hoyt DB. The use of hypertonic saline in the treatment of traumatic brain injury. J Trauma 2001; 50: 367-383.

32 Mann GE, Smaje LH, Yudilevich DL. Permeability of the fenestrated capillaries in the cat submandibular gland to lipid-insoluble molecules. J Physiol (Lond). 1979; 297: 335-354.

33 Holtz J. Peripheral circulation: fundamental concepts, comparative aspects of control in specific vascular sections, and lymph flow. In: Greger R, Windhorst U, editors. Comprehensive human physiology. Berlin: Springer; 1996. p. 1865-1915.
